# When giant coronary aneurysms masquerade as cardiac masses

**DOI:** 10.1093/ehjcr/ytag547

**Published:** 2026-07-30

**Authors:** Shokoufeh Hajsadeghi, Hamidreza Pouraliakbar, Reza Khosravi, Soroush Mostafavi

**Affiliations:** Research Center for Prevention of Cardiovascular Disease, Institute of Endocrinology & Metabolism, Iran University of Medical Sciences (IUMS), Shahid Hemmat Highway, Tehran 1449614535, Iran; Shaheed Rajaie Cardiovascular Medical and Research Institute, Iran University of Medical Sciences, Valiasr Ave., Niayesh Intersection, Tehran 1995614331, Iran; Department of Cardiology, School of Medicine, Hazrat-e Rasool General Hospital, Iran University of Medical Sciences (IUMS), Niyayesh St, Sattar Khan St, Tehran, Iran 1445613131; Department of Cardiology, School of Medicine, Hazrat-e Rasool General Hospital, Iran University of Medical Sciences (IUMS), Niyayesh St, Sattar Khan St, Tehran, Iran 1445613131

## Case Description

A 63 year old man with history of hypertension and dyslipidemia presented with a 2 month history of burning retrosternal chest pain and dyspnea, which had recently worsened.

**Figure 1:**
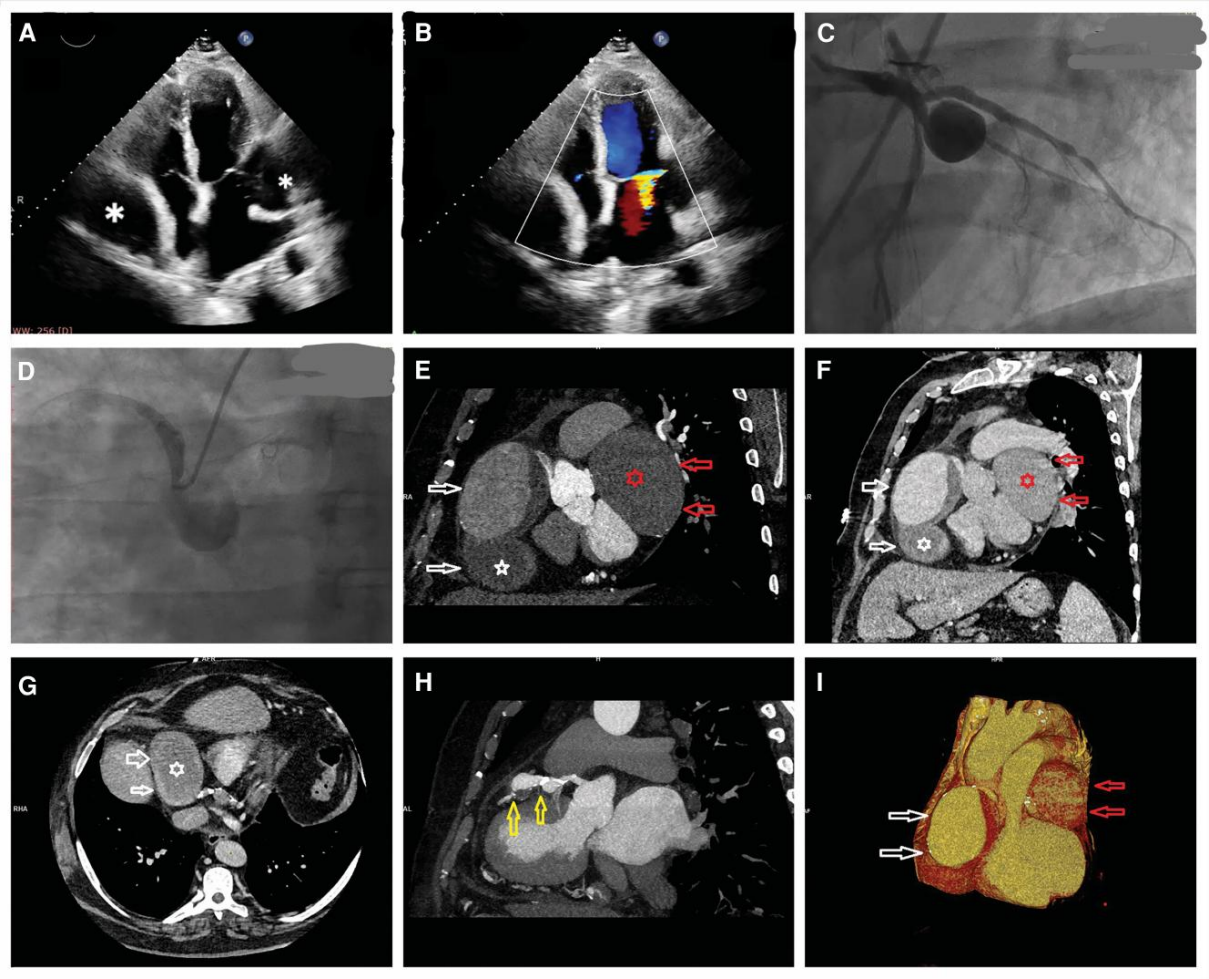
Echo-free masses (asterisks) adjacent to the left and the right atrium (*A* and *B*). Coronary angiography revealed three-vessel disease and aneurysms of the LAD, LCX (*C*) and RCA (*D*). Multidetector coronary computed tomography angiography (*E–I*) with multiplanar reconstruction of figures demonstrate the huge aneurysm of mid-to-distal portion of RCA with maximum diameter ∼80 mm (white arrows) and mural thrombosis (white stars), explaining the echo-free structure adjacent to the right atrium (*E–G*). Huge aneurysm of the proximal portion of LCX with maximum diameter ∼82 mm (red arrows) and contains thrombosis (red stars) (*E* and *F*). There are 4 different size saccular and fusiform aneurysms (maximum diameter 13.8 mm) with partial thrombosis in proximal to mid portion of LAD (yellow arrows) (*H*). The volume rendering reconstruction show aneurysms of RCA and LCX (*I*).

Transthoracic echocardiography revealed normal ejection fraction (55%) and a large (71 × 75 mm) echo-free extracardiac mass adjacent to the left atrium and another round (50 × 50 mm) echo-free structure adjacent to the right atrium (*Figure 1*, Panels *A* and *B*) (online video 1). Both lesions were best visualized in apical four-chamber view. Color Doppler study demonstrated no communication with the adjacent cardiac chambers. Contrast echocardiography was not performed. Their location and echocardiographic appearance raised concern for cardiac masses, with differential diagnosis including pericardial cysts, and extracardiac tumors.

The ECG showed no ischemic changes but patient had persistent typical chest pain. So we proceeded with invasive coronary angiography (CAG).

CAG demonstrated three-vessel disease with diffuse ectasia and aneurysms of the left anterior descending (LAD), left circumflex (LCX), and right coronary artery (RCA) (*Figure 1*, Panel *C* and *D*) (online video 2), but did not fully explain the echocardiographic findings.

Coronary computed tomography angiography (CCTA) (*Figure 1*, Panels *E–I*) revealed giant aneurysms of the proximal LCX, proximal-to-mid portion of LAD with mural thrombosis, and mid-to-distal RCA with layered thrombus. These aneurysmal segments corresponded anatomically to the echo-free masses seen on echocardiography.

The ischemic nature of the presentation and the presence of mural thrombosis necessitated the initiation of aspirin and warfarin. Due to diffuse involvement of all three coronary arteries with multiple giant aneurysms without obstructive culprit lesions, percutaneous coronary intervention (PCI) was not undertaken.

Surgical intervention is generally reserved for Kawasaki-related aneurysms or cases that are not amenable to PCI, neither of which applied to our patient.^1^

At 6 month follow-up, no chest pain or dyspnea was reported. Unfortunately the patient refused further work-up and imaging.

There was no history of systemic vasculitis, connective tissue disease, or Kawasaki disease. According to age, cardiovascular risk factors, and CAG findings, advanced atherosclerosis was considered the most probable underlying aetiology.^2^

The differential diagnosis of echo-free paracardiac lesions includes pericardial cysts, cardiac or extracardiac tumors, vascular anomalies and other cystic lesions of the mediastinum. Unlike CAG, which only demonstrates the contrast-filled lumen, CCTA accurately delineates the exact dimensions of the aneurysm, mural thrombi, and their anatomical relationship to adjacent structures, thereby reconciling discrepancies between echocardiographic and CAG findings and confirming the coronary origin of the lesions.^1^

These findings emphasize the pivotal role of multimodality imaging in differentiating giant coronary aneurysms from other echo-free paracardiac masses and avoiding inappropriate management.

Although coronary CT angiography confirmed the diagnosis in this case, cardiac magnetic resonance imaging could have provided complementary assessment of myocardial tissue characteristics, ventricular function, viability, and, if necessary, further prognostic features of suspicious cardiac masses.^1^

## Supplementary Material

ytag547_Supplementary_Data

## Data Availability

Data available on request.

## Notes

**Consent:** Written consent was obtained from the patient.
